# Improving service efficiency and throughput of cardiac surgery patients using Monte Carlo simulation: a queueing setting

**DOI:** 10.1038/s41598-022-25689-y

**Published:** 2022-12-08

**Authors:** Tayeb Mohammadi, Ghodratollah Roshanaei, Javad Faradmal, Majid Sadeghifar, Babak Manafi, Hossein Mahjub

**Affiliations:** 1 Department of Biostatistics, School of Public Health, Hamadan University of Medical Sciences, Hamadan, Iran; 2 Modeling of Noncommunicable Diseases Research Center, Hamadan University of Medical Sciences, Hamadan, Iran; 3 Department of Statistics, Faculty of Basic Sciences, Bu‐Ali Sina University, Hamadan, Iran; 4 Department of Cardiac Surgery, Faculty of Medical Sciences, Farshchian Heart Center, Hamadan University of Medical Sciences, Hamadan, Iran; 5 Research Center for Health Sciences, Hamadan University of Medical Sciences, Hamadan, Iran

**Keywords:** Cardiology, Health care, Medical research, Engineering, Mathematics and computing

## Abstract

Bed occupancy rate (BOR) is important for healthcare policymakers. Studies showed the necessity of using simulation approach when encountering complex real-world problems to plan the optimal use of resources and improve the quality of services. So, the aim of the present study is to estimate average length of stay (LOS), BOR, bed blocking probability (BBP), and throughput of patients in a cardiac surgery department (CSD) using simulation models. We studied the behavior of a CSD as a complex queueing system at the Farshchian Hospital. In the queueing model, customers were patients and servers were beds in intensive care unit (ICU) and post-operative ward (POW). A computer program based on the Monte Carlo simulation, using Python software, was developed to evaluate the behavior of the system under different number of beds in ICU and POW. The queueing simulation study showed that, for a fixed number of beds in ICU, BOR in POW decreases as the number of beds in POW increases and LOS in ICU increases as the number of beds in POW decreases. Also, based on the available data, the throughput of patients in the CSD during 800 days was 1999 patients. Whereas, the simulation results showed that, 2839 patients can be operated in the same period. The results of the simulation study clearly demonstrated the behavior of the CSD; so, it must be mentioned, hospital administrators should design an efficient plan to increase BOR and throughput of patients in the future.

## Introduction

Globally, life expectancy has increased during the last two decades which causes an increase in the elderly population^1^. One of the consequences of aging phenomenon is the increased prevalence of cardiovascular diseases (CVDs)^2,3^. According to the World Health Organization report, about 18 million deaths were attributed to CVDs in the year 2019, accounting for 32% of all global deaths^4^. Coronary artery disease (CAD), as the most common type of CVD, is one of the primary causes of disability and mortality worldwide^5–10^. Coronary artery bypass grafting (CABG) surgery is one of the most common procedures which is traditionally used to treat CADs around the world^11–13^. It has been reported patients who underwent CABG have longer survival compared with medically treated patients^14^. Also, It has been estimated that the number of CABG surgeries in the United States reaches to four hundred thousand annually^15^. The length of stay (LOS) and the bed occupancy rate (BOR) are the most important measures for evaluating the performance of hospital systems^16^. Intensive care units (ICUs), as a bottleneck after operating room, play an important role in spending recovery period of patients after surgery in hospitals, and there are expensive advanced medical equipment and specialized staff working there^17,18^. In the United States, about 6 million admissions to ICU occur in each year and it has been reported that the ICU costs accounted for approximately 15% of the total hospital costs^17^. Nowadays, managers, especially in the field of healthcare systems, are facing with serious challenges due to lack of enough resources, limited budget, shortage of equipment (e.g. beds) and professional manpower (e.g. nurse, surgeon etc.). So, they are seeking operational methods to reduce costs, increase efficiency and improve the quality of care in order to facilitate and timely access to services as well as enhancing patient satisfaction in the community^19,20^.

Hospital beds are one of the most valuable resources and optimizing use of them has been received more attention among researchers^21^. Based on a report in 2013, approximately 36,000 patients have been undergone cardiac surgery in the United Kingdom along with cost of about £300 million annually^22^. On the other hand, it has been reported that the scarcity of surgical and fiscal resources can lead to prolonged waiting times before operation particularly among patients who were candidate for CABG surgery. Lastly, it may increase the risk of death^23,24^. It is an interesting idea to view the behavior of the cardiac surgery department (CSD) as a dynamic system. Hence, in order to improve the throughput of patients, we can consider the system as a complex queueing system in which the servers are the beds in the post-operative ward (POW) and ICU. When examining these complex systems, it usually is very difficult and at times even impossible to obtain an analytical closed-form solution. So, one of the most common alternative methods to study this system is using the Monte Carlo simulation technique^25–27^. Due to complexity of systems and growing power of computers especially over the last two decades, the application of simulation models and queueing systems in healthcare area and real-world studies is increasing^28–31^. Several studies have been conducted in this field, for example, Muer Yang et al. used the queueing and simulation models simultaneously, to study the relationship between ICU beds and operating room in a cardiothoracic surgical ICU^29^. Bahadori et al. applied queuing theory and simulation technique to optimize the management of an outpatient pharmacy^32^. Saville et al. applied computer simulation to optimize the number of nursing staff allocated to each hospital ward^33^. Belciug and Gorunescu applied queueing modeling and simulation to a geriatric department of a hospital to improve hospital bed occupancy and resource utilization^34^. Marcon et al. proposed a computer simulation flow model to determine the minimum number of beds required in the post-anesthesia care unit^35^. Saadouli et al. used a stochastic optimization and simulation approach in order to optimize the resources in an orthopedic surgery department^36^. Azari-Rad et al. used discrete event simulation to reduce elective general surgery cancellations^37^. Also, El‐Darzi et al. used a discrete event simulation to model the flow of patients in an hospital geriatric department^38^. At last, Mahjub and Cox applied a computer program for Monte-Carlo simulation of a CSD^25^. Given the literature review, it is clear now why efficient management of cardiac surgery facilities is so important.

The BOR in Iranian hospitals was estimated at 57.8 percent in 2008, which has a significant difference with the global standard index (85–90 percent)^39^. On the other hand, a CSD is one of the most important parts of a hospital that is very expensive to equip and maintain. So, we chose a hospital, Farshchian Hospital as one of the most equipped heart hospitals in Iran, and focused on its heart surgery department. We were interested to know its maximum capacity via a simulation study to find out if there is any extra capacity to increase its performance.

So, the main objective of the present study is to observe the behavior of the system using a Monte Carlo queuing simulation to determine optimum number of beds in ICU and ward in a CSD.

## Material and methods

### Data and patients

This study was approved by the ethics committee of Hamadan University of Medical Sciences (Approved ethical code: IR.UMSHA.REC.1398.396), and all methods were carried out in accordance with relevant guidelines and regulations. Prior written informed consent was obtained from all the participants.

In this study, we collected data from computerized (electronic) medical records of 1999 patients who underwent cardiac surgery, including three categories: 1. CABG, 2. Aortic Valve Replacement, Mitral Valve Replacement and Tricuspid Valve Replacement and 3. Heart Septal Defects: Ventricular Septal Defect and Atrial Septal Defect, at the Farshchian Heart center in Hamadan, Iran, during a 26—month period, 800 days, from 22 December 2017 to 29 February 2020.

**Figure 1:**
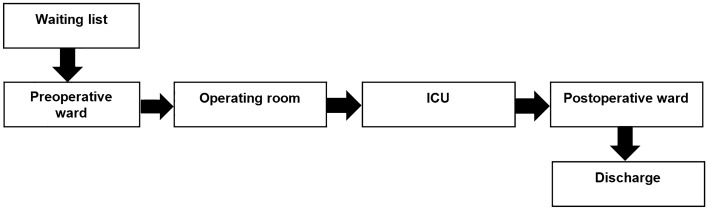
Demonstration of patient flow in the cardiac surgery department (CSD).

The patients who did not respond to conventional therapeutic procedures, in order to surgical consultation were referred to a cardiac surgeon by a cardiologist. After examination and observation of medical evidence by surgeons, eventually they may become candidates for cardiac surgery. The patients were generally classified into emergency (non-elective) and elective groups according to their clinical conditions. Emergency patients were operated earlier than elective patients without being discharged from the hospital. The elective patients entered into cardiac surgical waiting list to call for surgery according to turn schedule. In this center, there were four on-call surgeons to do cardiac surgery on their duties. Also, at present, the CSD act with 10 beds in ICU and 16 beds in POW. The general flow of patients and the possible events that occur in the CSD are as follows: First, patients arrive to a preoperative ward from the waiting list, then go to the operating room for operation, and afterward are transferred to the ICU for recovery, then move to POW, and finally discharged. This situation is portrayed in Fig. 1.

Each patient normally stays one or two days in preoperative ward; if there is an empty bed in ICU, she/he undergoes surgery and stays in ICU for at least one day. Depending on the patient’s clinical conditions, she/he has a different LOS in ICU and POW. Due to the fact that the number of deaths that occurred during and after surgery was very small and insignificant, we did not consider it in the simulation program. Since the unit of time is a day, and the events occur sequentially at discrete points of time, also, the simulation time is advanced simultaneously along with occurrence time of events, therefore, the system under study can be seen as a discrete-event system. Hence, we considered this type of simulation approach as discrete event simulation.

In some epidemic conditions such as coronavirus disease 2019 (COVID-19), in order to isolate patients, the admission of patients in CSD was affected and the POW was usually closed. Therefore, patients were discharged from the ICU after surgery. In system simulation, these conditions can be considered by keeping and prolonging each patient's stay in the ICU until discharge and zeroing the POW LOS. This situation can be implemented by adding a module (subroutine) to the simulation program.

In the simulation program, the exact path of Patients’ movements within the system and the LOS in each ward is kept tracked and controlled on a daily period. In the following, we list the details of the basic assumptions that were considered when developing the simulation program:

- 1. The LOS in the ICU and the POW was defined as the number of nights that a bed is occupied by each patient.
- 2. There are always some patients on the waiting list. In other words, there is an infinite supply of patients waiting to have cardiac surgery.
- 3. Elective patients were operated in order of admission times. (Known as the first-come, first-served rule in queueing theory).
- 4. Patient^’^s surgery and admission is performed on all weekdays, except Fridays and public holidays.
- 5. Patients can be discharged on any day of the week.
- 6. During a year, about 20% of the days were holidays (about 14% on Fridays and 6% on other public holidays). In fact, rather than actual dates of holidays, each day was simulated to be a holiday with a probability of 0.2.
- 7. In the queueing system, beds were the servers and patients were considered as the customers.
- 8. The order in which events are simulated in the system is for: a. A discharged patient, b. A patient who is leaving ICU to POW, c. A patient who is arriving from preoperative ward to ICU, d. Finally, admission of a new patient from waiting list.

In the Monte Carlo- based simulation method^40,41^, the first important step is finding the distribution functions of LOS (service time) in ICU and POW. In this study, at first, some common parametric distributions including Lognormal, Gamma, Weibull and Loglogistic were fitted to the LOS data in ICU and POW by using “actuar”^42^ and “fitdistrplus”^43^ packages in R software version 4.1.2, then, based on the highest log likelihood statistics, the best distributions for LOS in ICU and POW were chosen.

The bed blocking probability (BBP) was considered as the probability of a ready patient is not able to move from ICU to POW due lack of empty beds in POW. This probability was computed as the ratio of the number of patients who are not able to move from ICU to POW due lack of empty beds in POW to the total number of patients who are ready to move from ICU to POW.

The parameters for the fitted LOS distributions in the ICU and POW, the number of simulation days, and the number of beds in ICU and POW were given as input into the simulation program. After initialization, the system was simulated with 500 replications. The average and standard deviation (SD) of desired performance measures were calculated. The desired performance measures of our simulation study were, estimation of average BOR, LOS in ICU, throughput of patients in the system, and BBP. The simulation program code was written in Python programming software, version 3.9.5, along with installing and importing all necessary libraries^44^. GraphPad Prism software (version 6.0, GraphPad, San Diego, CA, USA) was also used.

## Results

### Descriptive results

**Figure 2:**
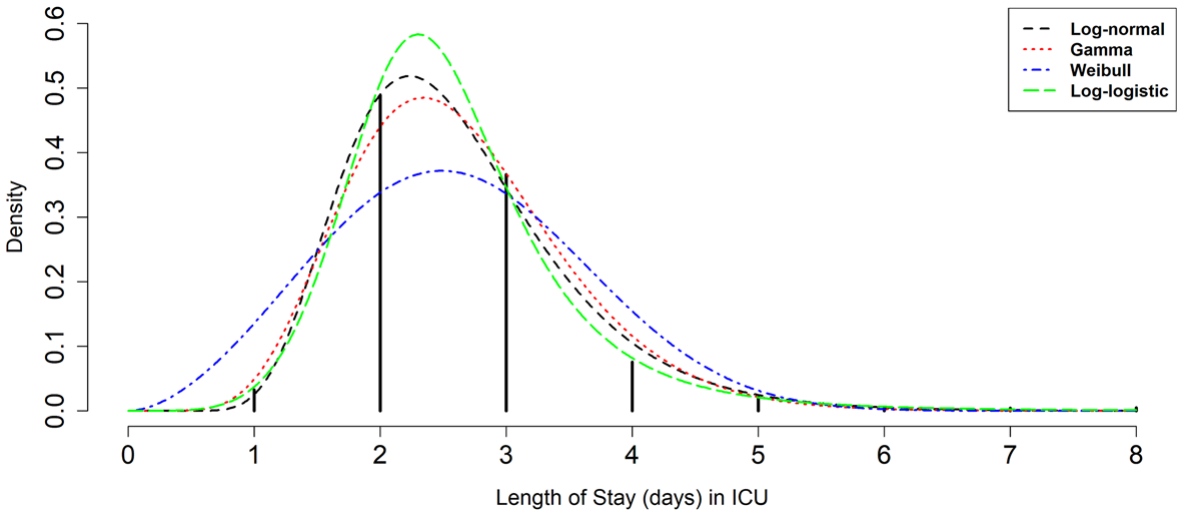
Length of Stay (LOS) in the ICU after operation based on empirical LOS distribution and fitted parametric distributions.

**Figure 3:**
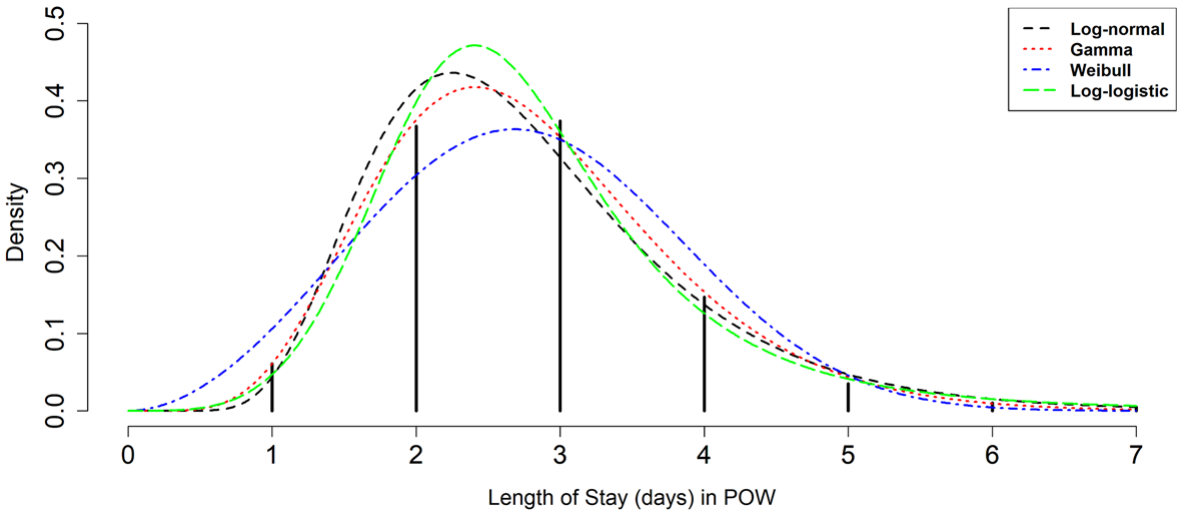
Length of Stay (LOS) in the post operative ward (POW) based on empirical LOS distribution and fitted parametric distributions.

The mean (SD) of LOS in ICU and POW were 2.62 (0.94) and 2.78 (1.01) days, respectively. Figures 2 and 3 show visually empirical LOS distribution based on the real data and some fitted parametric distributions to LOS (days) in ICU and POW, respectively. As can be seen, most patients spend two (48.9% and 36.8%) or three (36.6% and 37.4%) days in ICU and POW, respectively. Based on the highest log-likelihood values, the best fitted distribution for LOS in ICU was the Log logistic distribution (*shape* parameter *β* = 5.55 and *scale* parameter *α* = 2.46) and for POW was the Gamma distribution (*shape* parameter *β* = 7.53 and *scale* parameter *α* = 0.37).

### Simulation results

**Figure 4:**
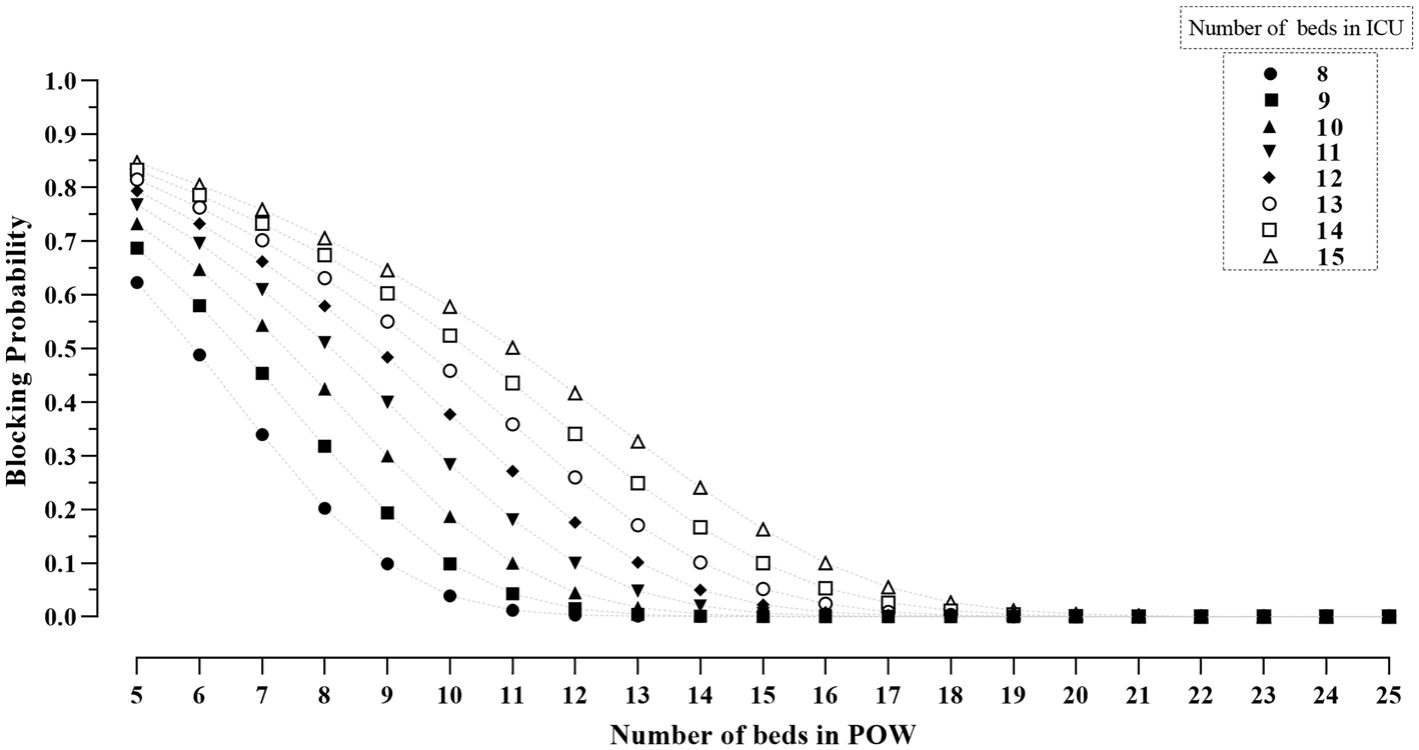
The bed blocking probability (BBP) at post operative ward (POW) for different number of beds in ICU and POW.

**Figure 5:**
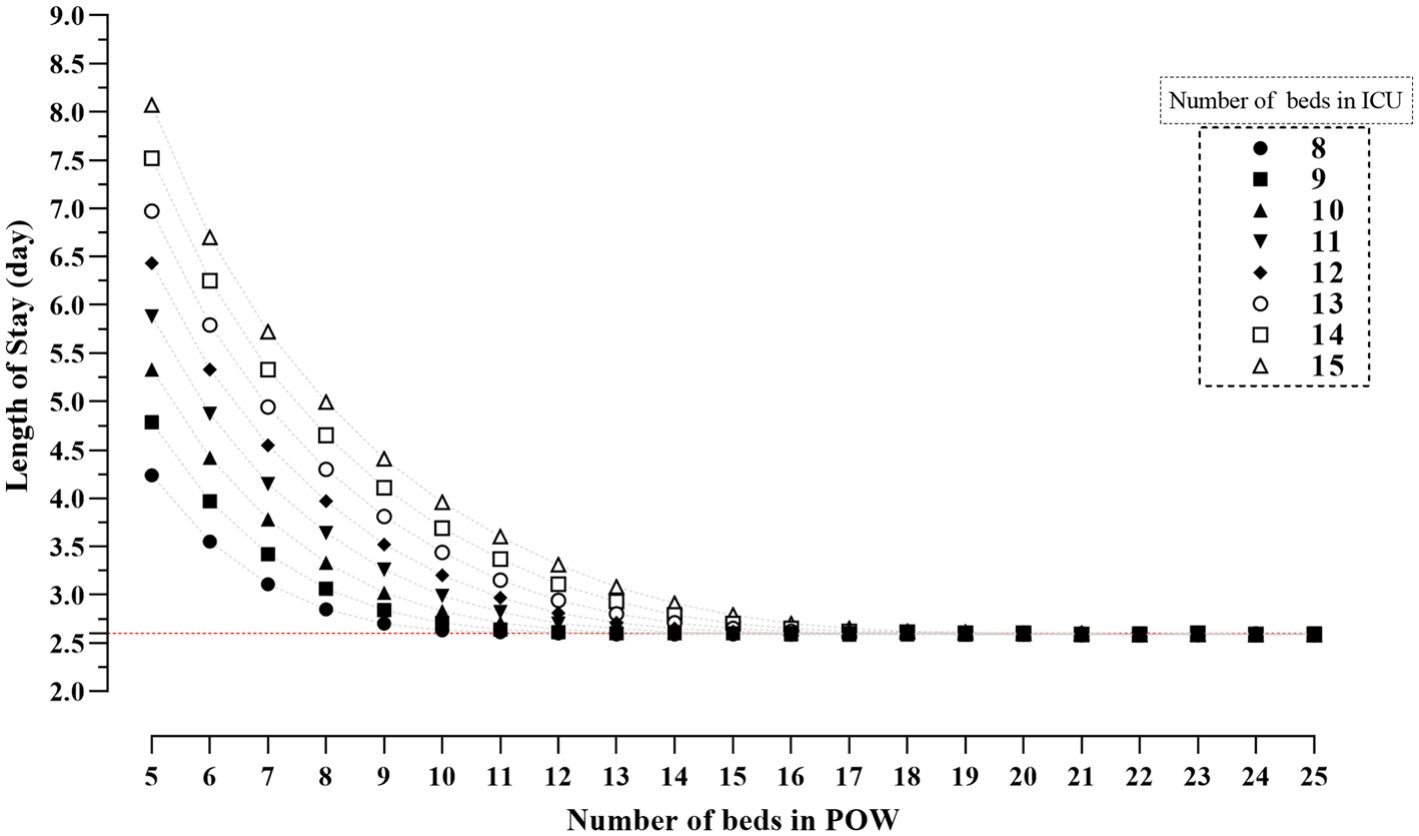
Length of Stay (LOS) in ICU after operation for different number of beds in ICU and post operative ward (POW).

Tables 1 and 2, report results of the simulation study regarding how BOR in POW and patient throughput vary as a function of number of beds in ICU and beds in POW. Also, the trends of BBP and LOS in ICU for different numbers of beds in ICU and POW are shown in Figs. 4 and 5, respectively.

**Table 1:** Bed occupancy rate (%) in post operative ward (POW) for different number of beds in ICU and POW.

No. of beds in Post Operative Ward	No. of beds in ICU							
	8	9	10	11	12	13	14	15
	Mean (SD)	Mean (SD)	Mean (SD)	Mean (SD)	Mean (SD)	Mean (SD)	Mean (SD)	Mean (SD)
5	100 (5)	100 (4)	100 (3)	100 (3)	100(3)	100 (3)	100 (3)	100 (3)
6	98 (7)	99 (5)	100 (4)	100 (3)	100 (3)	100 (3)	100 (3)	100 (3)
7	95 (11)	98 (8)	99 (5)	100 (4)	100 (4)	100 (3)	100 (3)	100 (3)
8	91 (14)	95 (11)	98 (8)	99 (6)	100 (4)	100 (4)	100 (3)	100 (3)
9	85 (16)	91 (14)	95 (11)	98 (8)	99 (6)	99 (5)	100 (4)	100 (4)
10	78 (17)	85 (16)	91(13)	95 (10)	98 (8)	99 (6)	99 (5)	100 (4)
11	71 (17)	80 (17)	86 (15)	92 (13)	95 (10)	97 (8)	99 (6)	99 (5)
12	66 (16)	74 (16)	81 (16)	87 (15)	92 (13)	95 (10)	97 (8)	99 (6)
13	61 (15)	68 (16)	75 (16)	82 (16)	88 (14)	92 (12)	95 (10)	97 (8)
14	56 (14)	63 (15)	70 (15)	77 (16)	83 (15)	88 (14)	92 (12)	95 (10)
15	53 (13)	59 (14)	66 (15)	72 (15)	78 (15)	84 (15)	89 (13)	92 (12)
16	49 (12)	56 (13)	62 (14)	68 (14)	74 (15)	80 (15)	85 (14)	89 (13)
17	46 (11)	52 (12)	58 (13)	64 (14)	70 (14)	75 (15)	81 (15)	85 (14)
18	44 (11)	49 (11)	55 (12)	60 (13)	66 (14)	71 (14)	77 (14)	81 (14)
19	42 (10)	47 (11)	52 (12)	57 (12)	62 (13)	67 (14)	73 (14)	78 (14)
20	40 (10)	44 (10)	49 (11)	54 (12)	59 (12)	64 (13)	69 (13)	74 (14)
21	38 (9)	42 (10)	47 (10)	52 (11)	56 (12)	61 (12)	66 (13)	71 (13)
22	36 (9)	40 (9)	45 (10)	49 (11)	54 (11)	58 (12)	63 (12)	67 (13)
23	34 (8)	39(9)	43 (10)	47 (10)	52 (11)	56 (11)	60 (12)	64 (12)
24	33 (8)	37 (9)	41 (9)	45(10)	49 (10)	53 (11)	58 (11)	62 (12)
25	32 (8)	36 (8)	40 (9)	43 (9)	47 (10)	51 (10)	55 (11)	59 (11)

*SD* standard deviation.

**Table 2:** The throughput of patients during 800 days for different number of beds in ICU and post operative ward (POW).

No. of beds in Post Operative Ward	No. of beds in ICU							
	8	9	10	11	12	13	14	15
	Mean (SD)	Mean (SD)	Mean (SD)	Mean (SD)	Mean (SD)	Mean (SD)	Mean (SD)	Mean (SD)
5	1431 (14)	1436 (14)	1437 (14)	1435 (14)	1436 (15)	1436 (14)	1436 (14)	1436 (15)
6	1698 (15)	1715 (16)	1722 (16)	1723 (16)	1723 (15)	1723 (15)	1723 (16)	1724 (16)
7	1920 (14)	1976 (15)	1997 (16)	2008 (17)	2010 (17)	2009 (17)	2011 (17)	2011 (16)
8	2087 (14)	2193 (15)	2253 (16)	2280 (17)	2291 (17)	2297 (17)	2294 (18)	2297 (19)
9	2188 (17)	2356 (15)	2467 (17)	2529 (18)	2562 (18)	2576 (19)	2581 (19)	2585 (19)
10	2240 (18)	2459 (18)	2626 (18)	2740 (17)	2807 (19)	2843 (19)	2859 (18)	2866 (20)
11	2261 (20)	2516 (20)	2731 (20)	2899 (18)	3012 (19)	3083 (18)	3123 (19)	3142 (21)
12	2269 (20)	2544 (21)	2794 (21)	3007 (21)	3169 (19)	3286 (20)	3359 (19)	3402 (23)
13	2271 (21)	2553 (23)	2823 (22)	3069 (22)	3280 (23)	3442 (21)	3559 (21)	3637 (22)
14	2273 (20)	2554 (22)	2836 (23)	3102 (25)	3346 (23)	3552 (21)	3716 (22)	3833 (22)
15	2273 (21)	2555 (23)	2839 (22)	3113 (23)	3381 (24)	3622 (25)	3826 (24)	3988 (23)
16	2272 (20)	2556 (22)	2839 (23)	3122 (25)	3398 (25)	3661 (27)	3898 (25)	4101 (26)
17	2271 (20)	2556 (22)	2840 (25)	3122 (25)	3403 (27)	3681 (28)	3939 (27)	4174 (26)
18	2270 (20)	2554 (21)	2840 (23)	3124 (25)	3407 (26)	3687 (29)	3960 (28)	4217 (29)
19	2273 (20)	2556 (22)	2840 (24)	3123 (25)	3407 (26)	3690 (28)	3969 (30)	4242 (31)
20	2273 (21)	2555 (22)	2840 (24)	3124 (25)	3409 (26)	3690 (30)	3975 (29)	4252 (29)
21	2271 (20)	2556 (23)	2839 (23)	3125 (26)	3409 (27)	3691 (28)	3976 (30)	4258 (32)
22	2274 (20)	2558 (22)	2839 (23)	3122 (26)	3409 (27)	3693 (29)	3976 (30)	4258 (32)
23	2272 (20)	2555 (22)	2837 (23)	3125 (25)	3407 (28)	3692 (28)	3974 (30)	4260 (33)
24	2272 (21)	2556 (21)	2841 (24)	3124 (25)	3410 (28)	3691 (30)	3975 (28)	4260 (32)
25	2272 (20)	2556 (22)	2841 (22)	3121 (25)	3407 (28)	3693 (29)	3977 (29)	4260 (33)

*SD* standard deviation.

A summary of findings from the simulation study are as follows:

1. For a fixed number of beds in ICU, BOR in POW decreases as the number of beds in POW increases.
2. For a fixed number of beds in ICU, LOS in ICU increases as the number of beds in POW decreases.
3. The throughput of patients increases as the number beds in ICU and POW increases.
4. The BBP decreases as the number of beds in POW increases.

As Fig. 4 shows when there are 10 beds in ICU and 5 beds in POW the BBP is 0.73 which approaches zero when the number of beds in POW exceeds 11. Finally, as Fig. 5 demonstrate, as the number of POW in beds increases, the LOS in ICU stabilized at 2.6 days which is compatible with the empirical average of LOS in ICU that was 2.66 days.

Table 3 shows the simulation results when there are several months of COVID-19 restrictions throughout the year. For example, if the restriction is applied for a quarter of the days of the year, three months, with 10 beds in the ICU and 16 beds in the POW, the patient throughput drops from 2839 to 2251. In general, as the percentage of COVID-19 restrictions increases, fewer patients undergo surgery during the 800 study days.

**Table 3:** The throughput of patients during 800 days for **16** beds in POW and different number of beds in ICU during pandemic restrictions.

Restriction time per year(month)	No. of beds in ICU		
	8	10	15
	Mean (SD)	Mean (SD)	Mean (SD)
1	2075 (34)	2593 (41)	3801 (50)
2	1930 (33)	2412 (44)	3578 (57)
3	1804 (35)	2251 (44)	3358 (60)
4	1692 (32)	2114 (43)	3161 (58)

*POW* post-operative ward, *ICU* intensive care unit.

## Discussion

The application of simulation models is broadly common in healthcare systems to improve hospital resources management. Hospitals as one of the most important units of the healthcare systems, accounting for 50 to 80 percent of total health care costs. Because of that, their optimal and efficient management has become one of the challenges of governments and health sector managers in recent decades^45^. Many studies have used analytical and simulation methods to solve some of the problems in this field^20,30,32,36,38,46,47^. Whatever the complexity of the systems increases, analytical models are not responsive because they fail to capture the details. For this reason, one of the basic strategies for overcoming these limitations in the study of dynamic and complex systems is use of very flexible simulation methods^44^. In this study, we have developed a discrete-event simulation model of a CSD of a hospital in Iran. Therefore, we used a Monte Carlo simulation approach to mimic the behavior of patients in a CSD as a dynamic system; So, some of the performance measures including, the mean BOR, LOS, throughput of patients, and BBP under different number of beds in ICU and POW were estimated. The findings of our study showed, as the number of beds in POW increases, so does the throughput and also decreases queueing time in the ICU. Mahjub and Cox also used a computer program based on Monte Carlo simulation to estimate the BOR and throughput of patients in a CSD in England^25^; There are some similarities and differences between our research with their work; In both, the beds in ward and ICU were the servers and patients are considered as the customers. In their study, only BOR and throughput of patients were calculated, but we also estimated the mean LOS in ICU and probability of being blocked (BBP) in POW. They computed the mean required time for throughput of 500 patients for varying numbers of beds in ward and ICU, but we estimated the mean (SD) of number of discharged patients (throughput) during 800 days for different number of beds in ICU and POW. So, from a managerial and Cost-effectiveness view, the average throughput with 8 beds in ICU and 11 beds in POW is approximately the same as 10 beds in ICU and 8 beds in POW.

We used Python programming due to some of its key advantages, including easy expansion, code readability, ease of use, high flexibility, user friendly, object-oriented capability, high computational speed and etc.^48^. Gorunescu et al. used a classical queueing theory to determine the optimal number of beds, in which hospital beds had the role of servers and LOS as service time using phase-type distributions; however, no simulation methods were used in their study^49^. In the present study, we used Log logistic and Gamma distributions for LOS (service times) in ICU and POW, respectively. Also, according to simulation findings, when the number of beds in POW exceeds a certain limit, the BBP begins to converge to zero, and as a result, patients will not have to wait in ICU.

In this study, beds play the role of servers, so the main purpose was to determine the optimal number of beds, especially in ICU, because the cost of providing and adding a bed in ICU, as a bottleneck resource, is much higher than in POW. Since financial resources are limited, it is important to be able to justify and satisfy hospital managers before spending money and purchasing new equipment. They are also convinced that the new changes, the optimal allocation of resources and beds will lead to long-term financial profits; and also, an increase in hospital revenue in the future.

One of the advantages of this study is use of very powerful and popular programming software, Python^44^. In many simulation studies, the application of Python software is expanding due to its high flexibility in modeling complex systems and its ability to consider real-world scenarios. Although various simulation software is now designed and readily available in academic and industrial settings^50,51^, however, choosing the appropriate one is a difficult task because many of them are not suitable or even applicable in some complex situations. Also, it is practically impossible for researchers to develop them in new situations. Another main strength of our study is considering annual holidays in the simulation program, so that about 20% of the days of the year, including 14% Fridays and 6% of public holidays, no surgery was performed. In general, our simulation program is precisely set up based on the realities that occur in the CSD.

To check the accuracy of the results of LOS modeling (Log logistic and gamma distributions), the empirical distributions of LOS were also used for simulation. Since the results of the two approaches were close to each other, it can be concluded that the models were chosen correctly and the simulation results are valid.

There are some limitations in this study. As mentioned earlier, a number of assumptions were taken into account during modeling and programming. We implicitly assumed that there was no limit on number of surgeons or operating rooms. So, as soon as an empty bed is in ICU, a patient would undergo surgery. However, not only the number of surgeons is limited, four surgeons in this study, the number of surgeries performed by each surgeon is also limited maximum of two surgeries per day.

In future studies, this limitation can be addressed by considering the surgeons and beds in the role of servers as well. Furthermore, taking into account average total cost of staying in the system may also help to study this system more precisely.

In the present study, deaths were not considered in simulation program; this is not a disadvantage of the study, because the number of deaths occurring during or after the operation was very low; but for future research, if the percentage of postoperative deaths is high, it is a good idea to consider different scenarios of deaths in simulation program to better understand the behavior of the system.

In this research, patients who were candidates for cardiac surgery, were grouped into two classes; non-emergency and emergency, and priority must be given to the latter group, but we have assumed that all patients have the same priority and undergo surgery in the order of admission. According to a report of Iranian hospitals affiliated to the Ministry of Health and Medical Education of Iran, the BOR in Iranian hospitals in 2008 was estimated at 57.8 percent, which shows a significant difference with the global standard index (85–90 percent)^39^. This in line with our study that confirms with the current facilities, equipment, and medical personnel of investigated system, i.e. 10 and 16 beds (sometimes plus two extra beds) in ICU and POW, respectively, 1999 cardiac surgeries were performed during 800 days. But the results of the simulation program showed that with the same conditions, 2839 patients are expected to undergo surgery. In other words, the hospital system, with its current facilities, equipment and manpower, operates approximately 43% less than its actual efficiency. As already mentioned, the empirical average LOS in ICU was 2.66 days, and the simulation results also confirm that, as the number of POW beds increased, the LOS in ICU stabilized at 2.6 days.

The simulation program we have extended in this study can also be applied in other areas.

If during the year, the admission of patients is affected by the restrictions of the COVID-19 or other epidemics, these conditions can be considered in the simulation program. In our study, as previously mentioned, this situation is allowed by prolonging each patient’s ICU stay until discharge and setting POW LOS to zero in the simulation program. For instance, based on the simulation results, with 10 beds in the ICU and 16 beds in the POW, if we assume 4 months of restrictions per year, the throughput of patients is reduced from 2839 to 2114 during 800 days, a 25.5% reduction. On the other hand, based on the raw data, the average number of monthly surgeries before and after the covid epidemic was 66.78 and 51.26, respectively, which means it has decreased by about 23%. Therefore, the hospital's performance also confirmed that after the COVID-19 epidemic, patient admissions have decreased by almost the same proportion.

Since this simulation program depends on the LOS distribution of patients in the POW and ICU, it can be used for other diseases where patients are admitted to one or two consecutive wards. However, this program can also be extended to three or more consecutive sections.

Improving the efficiency, throughput, optimization of hospital system facilities, balancing between the supply of services by the hospital and also the demand for services by patients is a win–win approach. If waiting times would be shorter, patients will be able to access services faster. So, hospitals with proper planning for the future, have a strategy for optimal use of resources and its allocation; therefore, it would prevent the pressure of congestion and increasing demand.

In conclusion, the results of this simulation study were able to show behavior and performance measures of the system by considering different numbers of beds in ICU and ward clearly. Therefore, according to the simulation results, hospital managers can not only make optimal use of available resources and increase system efficiency, but would also be able to provide accurate planning for the future.

## Data Availability

The data that support the findings of this study are available from Hossein Mahjub (Email: mahjub@umsha.ac.ir) but restrictions apply to the availability of these data, which were used under license for the current study, and so are not publicly available. Data are however available from the authors upon reasonable request and with permission of the corresponding author.

## Abbreviations

BOR: Bed occupancy rate
ICU: Intensive care unit
POW: Post-operative ward
LOS: Length of stay
BBP: Bed blocking probability
CSD: Cardiac surgery department
CVD: Cardiovascular disease
CAD: Coronary artery disease
CABG: Coronary artery bypass grafting

## Code availability

The codes that support the findings of this study are available from Hossein Mahjub (Email: mahjub@umsha.ac.ir) but restrictions apply to the availability of these codes, which were used under license for the current study, and so are not publicly available. Codes are, however available from the authors upon reasonable request and with permission of the corresponding author.
